# Differentiating between regulation and hunting as conservation interventions

**DOI:** 10.1111/cobi.13211

**Published:** 2018-10-24

**Authors:** Adrian Treves, Kyle A. Artelle, Paul C. Paquet

**Affiliations:** ^1^ Nelson Institute for Environmental Studies University of Wisconsin‐Madison 550 North Park Street Madison WI 53706 U.S.A.; ^2^ Raincoast Conservation Foundation P.O. Box 2429 Sidney British Columbia V8L 3Y3 Canada; ^3^ Department of Geography University of Victoria P.O. Box 1700, Stn CSC Victoria British Columbia V8W 2Y2 Canada

**Figure 1:**
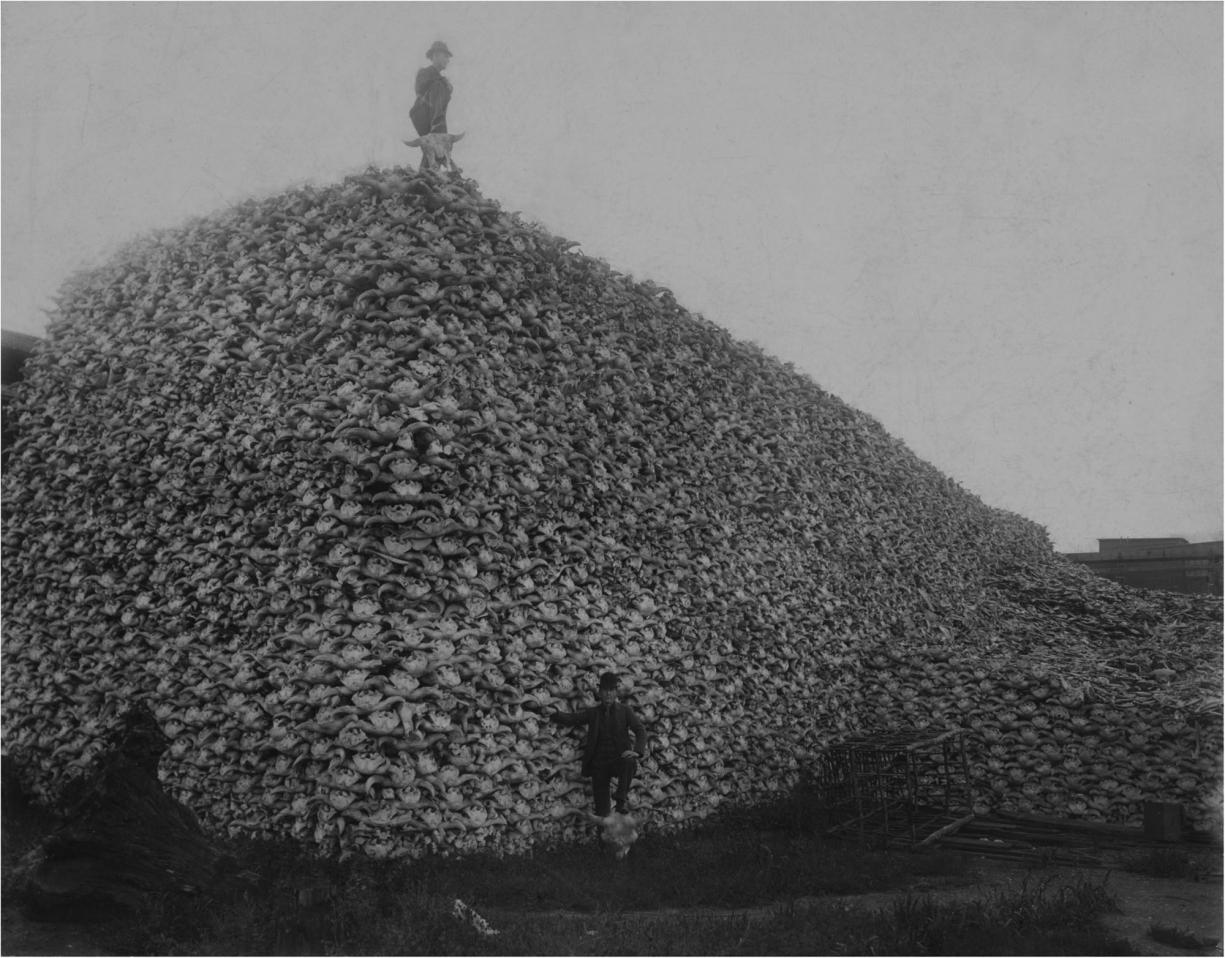
A pile of American bison skulls (mid‐1870s) waiting to be ground for fertilizer. Public domain photo (credit: https://en.wikipedia.org/wiki/Market_hunters#/media/File:Bison_skull_pile_edit.jpg).

Preventing extinction requires correct identification of major threats and effective interventions to abate them (Salafsky & Margoluis 2003; Sutherland et al. 2004). If the scientific community wants the world to heed warnings of ecosystem collapse (Ripple et al. 2017), it should be aware of past warnings and current misunderstandings. A century ago, similar alarms sounded over extinctions of wild animals taken for commercial meat markets (Roosevelt 1916). The near extinction of American bison (*Bison bison*) and other populations that were averted in the early 20th century provides useful contemporary lessons (Fig. 1). Then, overhunting threatened the persistence of multiple species, and the public‐policy intervention replaced unregulated commercial extraction with strict regulatory systems. Regulatory systems seem to have saved many wild animal populations from extinction by regulating methods and limiting participants and quantities taken by hunters and trappers. Yet, this view that regulation saved wild animals of western nations is persistently misrepresented and replaced in the scientific and management literature by an interpretation that hunting itself was the intervention.

The misrepresentation of the history is that the act of hunting, rather than regulation of hunting, saved commercial species from extinction. This misrepresentation was illustrated recently in a 19,000 word review aimed at “[f]inding effective ways of conserving large carnivores …” (Redpath et al. 2017). In this article, 19 prominent conservation scientists wrote, “…many predator populations thrive in the presence of hunting/trapping programs (hereafter just referred to as hunting) supported by local people…” (Redpath et al. 2017:2158). Without evidence that the populations are thriving, the authors condense hunting and trapping programs into simply *hunting* without considering permits, regulations, and enforcement and imply carnivores thriving with hunting is not unusual. Going back decades, one finds agencies and prominent institutions advocating hunting as a conservation intervention (Clark & Milloy 2014). For example, assertions that hunting is an effective conservation intervention in and of itself, without accompanying evidence of positive outcomes for the hunted populations, have been published or promoted by The International Union for the Conservation of Nature, The Wildlife Society, the Association of Fish and Wildlife Agencies, the Western Association of Fish and Wildlife Agencies, and the Wildlife Management Institute (Jackson 1996; Batcheller et al. 2010). Similar claims are made by academics penning titles, such as, “Why Lions Need to Be Hunted” (Howard 1988) or promoting trophy hunting generally (Di Minin et al. 2016). To be clear, we are not disputing the common and well‐substantiated claim that hunters and their organizations have contributed financially and through other indirect means to conservation (Holsman 2000). Nor is the problem we detect one of advocacy—all people prefer asking and answering certain questions and interpreting data in a particular way. Instead, we discuss how the lack of evidence supporting that advocacy misrepresents the intervention that protected animal populations in the past.

Hunting never directly saves the targeted animal. To our knowledge, there is no evidence that hunting has ever saved an animal population or species from extinction. By contrast, restrictions on hunting have certainly stemmed extinctions and extirpations (Wilcove 1999). These superficially obvious statements help to point the way to scientific evaluation of hunting as a conservation intervention. Specifically, hunting alone could only indirectly protect nontarget individual animals (Treves 2009). The conservation community needs incisive experiments to disentangle the hypothesis that hunting itself protects animals from the competing hypothesis that regulating hunting protects animals. No one to our knowledge has tested whether regulation or another aspect of modern hunting or trapping programs was the effective intervention in the early 20^th^ century. Was overexploitation by hunters and trappers prevented by the enforcement of quotas and bag limits or prevented by other factors related to organized hunting? Asserting that an action is an effective conservation tool without scientifically evaluating population‐level outcomes of that action, risks misleading the public and policy makers. The history of fisheries contains many such examples (Finley 2011). By analogy, scientists would cry foul if public health organizations touted eating to fight cancer, rather than touting a healthful diet (i.e., regulated eating). Touting hunting rather than regulated hunting can create a risky misconception. As Platt (1964) predicted, scientific fields in which researchers do not effectively identify and test opposing hypotheses will advance slowly, if at all. Only when claims about hunting are framed as opposing hypotheses will the field progress and the many claims about hunting as a conservation tool be falsifiable.

We see 3 pernicious consequences of omitting regulation from scientific treatments of conservation interventions. First, a lack of transparency about regulation prevents the objective evaluation of it as a help or hindrance to conservation efforts. For example, some might believe that regulation saved public hunting itself because a society might have banned all hunting when commercialization threatened the public's wildlife. Others might believe that regulation is a hindrance to hunting as a conservation instrument. By omitting mention of regulation, the implicit notion advances that regulation is unnecessary. Indeed, one must beware of omitting regulation from the narrative about hunting as a conservation intervention, especially given the potential for financial conflicts of interest created by powerful, moneyed interests seeking unlimited exploitation.

**Figure 2:**
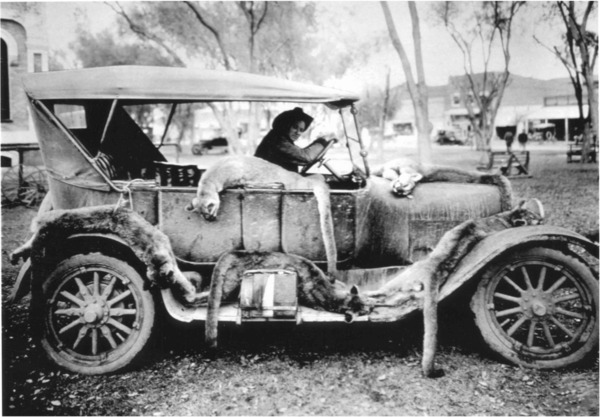
Cougars killed for market. Public domain photo (credit: https://upload.wikimedia.org/wikipedia/commons/3/30/Market_hunting_of_cougars.jpg).

That leads us to the second pernicious consequence of discounting regulation. When authorities ignore or underemphasize the importance of regulation, perpetrators of environmental crime, such as poachers, may feel emboldened or immune to prosecution. This idea was seemingly advocated by Kaltenborn and Brainerd (2016), who contend poaching acts as a release for rural resentment over national restoration of controversial wildlife. Treves et al. (2017a) reviewed 4 other cases in which inaccurate measurement of poaching led governments to downplay the major threat to endangered gray wolves (*Canis* spp.). Predators in particular seem to be targets for the idea that hunting itself is a conservation intervention (Fig. 2); the common hypothesis is that predator populations benefit indirectly when people kill a minority of them because then people tolerate the survivors better or revenue flows to direct conservation (Loveridge et al. 2007; Treves 2009; Treves & Bruskotter 2014; Chapron & Treves 2017; Macdonald et al. 2017).

The third pernicious consequence of forgetting the importance of regulation relates to the paucity of evidence about how regulated hunting works to prevent local extinctions. Given this paucity, our criticism of hunting as conservation might be seen as opposition to hunting itself. We do not, however, view hunting as incompatible with conservation. Confusing our work with antihunting advocacy would once again confuse hunting with the scientific evaluation of its effectiveness for protecting the hunted population.

To prevent extinctions, scientists must identify interventions that improve outcomes for populations. Decision makers must be transparent in their value judgments about human activities they permit (Treves et al. 2017b) and the evidence they use to allocate natural resources (Artelle et al. 2018; Batavia et al. 2018). Failure may contribute to ongoing extinctions and the erosion of public confidence in science.
